# New insights of P2X7 receptor signaling pathway in alveolar functions

**DOI:** 10.1186/1423-0127-20-26

**Published:** 2013-05-01

**Authors:** Amarjit Mishra

**Affiliations:** 1 National Institute of Health, 10 Center Dr, Bldg No. 10, Bethesda, MD 20892, USA

## Abstract

Purinergic P2X7 receptor (P2X7R), an ATP-gated cation channel, is unique among all other family members because of its ability to respond to various stimuli and to modulate pro-inflammatory signaling. The activation of P2X7R in immune cells is absolutely required for mature interleukin -1beta (IL-1beta) and IL-18 production and release. Lung alveoli are lined by the structural alveolar epithelial type I (AEC I) and alveolar epithelial type II cells (AEC II). AEC I plays important roles in alveolar barrier protection and fluid homeostasis whereas AEC II synthesizes and secrete surfactant and prevents alveoli from collapse. Earlier studies indicated that purinergic P2X7 receptors were specifically expressed in AEC I. However, their implication in alveolar functions has not been explored. This paper reviews two important signaling pathways of P2X7 receptors in surfactant homeostatsis and Acute Lung Injury (ALI). Thus, P2X7R resides at the critical nexus of alveolar pathophysiology.

## Review

Over the last two decades, a total of 19 different purinergic receptor subtypes (including 7 P2X receptors, 8 P2Y receptors, and 4 adenosine receptors) that can recognize extracellular ATP and adenosine have been cloned and characterized [1]. In addition, several families of ectonucleotidases that hydrolyse ATP to ADP, AMP and adenosine have been found [2]. These distinct sets of purinergic receptors and ectonucleotidases are expressed on the cell surface of the different mammalian cells and regulate cellular activities through cell-type specific purinergic signaling systems [3,4].

Controlled ATP release from intact cells was first identified in neurons [5]. ATP is also released from non-neuronal cells through vesicular transport [6]. Additional mechanisms for ATP release has been reported including release through stretch-activated channels, voltage-dependent and multi-channel anion transporter or permeases [7], cystic fibrosis transmembrane conductance regulator (CFTR) [8], and P2X7 receptor associated connexin and pannexin hemichannels [9]. ATP release from mouse neutrophil occurs through connexin-43 hemi channels [10].

Extracellular ATP has two fates before being degraded by the ectonucleotidases. The released ATP either acts on the purinoceptors of the same cell (autocrine) or the neighboring cells (paracrine). Autocrine signaling through the purinergic receptors regulates the neutrophil chemotaxis via ATP release from polarized neutrophil in response to chemotactic mediators [11]. The activated T cells also induce the release of ATP through pannexin 1 channels. These hemichannels translocate with P2X receptors to the immune synapse, where they promote Ca^2+^ influx and cell activation through autocrine purinergic signaling [12,13]. The activation of purinergic receptors in immune cells can elicit either positive or negative feedback mechanisms and thus tightly regulate immune responses.

Paracrine purinergic signaling regulates a wide range of physiological process, including immune cell functions [14,15]. ATP released from damaged or stressed host cells serves as an important function in the recognition of ‘danger signals’ and guides phagocytes to inflammatory sites. Thus promotes clearance of damaged and apoptotic cells [16]. In response to damage-associated molecular patterns (DAMPs) and pathogen-associated molecular patterns (PAMPs), activation of inflammasome and the subsequent release of interleukin-1β (IL-1β) require purinergic singnalling. In the cytoplasm nucleotides are concentrated in the micomolar or even millimolar level, while the extracellular concentration is extremely low, usually in the nanomolar range [17]. ATP is rapidly released upon damage of plasma membrane and diffuses throughout the pericellular space and bind to specific receptors expressed by virtually all immune cells [18]. Diffusion of nucleotides is drastically controlled via degradation by ecto-nucleotidases expressed on the plasma membranes of most cells. Rapid metabolism of extracellular ATP generates the anti-inflammatory metabolite adenosine and terminates the alert-signal to a checkpoint [19].

### P2X7 receptor and inflammation

P2X7 receptors are expressed primarily on the cells of hematopoietic lineage. The distribution of P2X7 receptor has been studied by permeability and RT-PCR analysis in cultured monocytes/macrophages [20], phagocytes [21], dendritic cells [22], T lymphocytes [23], B lymphocytes [24], mast cells [25] and eosinophils [26]. P2X7 receptor has also been identified in fibroblasts, endothelial cells and epithelial cells [27,28]. Our laboratory has reported previously specific expression of P2X7 receptors in alveolar type I epithelial cells (AEC I) [29].

IL-1β and IL-18 are pro-inflammatory cytokines that requires processing by interleukin converting enzyme (ICE, also known as caspase-1) at specific aspartic residues for mature molecule production. Activation of P2X7 receptor on human macrophages triggers the release of these two cytokines [30-32]. P2X7 receptor is also required for inflammasome assembly and caspase activation [33]. Studies on P2X7 receptor knock-out mice have shown that absence of P2X7 receptor leads to an inability to release IL-1β in response to ATP stimulation from peritoneal macrophages [34]. P2X7 null mice therefore have impaired cytokine signaling cascade *in vivo*. This suggests that P2X7 receptor activation provides signals for maturation and release of IL-1β and initiation of a cytokine cascade.

The unprocessed and mature form of IL-1β was found in the shed microvesicles [35]. P2X7–mediated microvesicle formation and shedding might be a crucial pathway for secretory protein release from cytoplasm. L-selectin (CD62L; a C-type lectin) and CD23 (low affinity IgE receptor) are involved in the adhesive interaction and rolling behavior of lymphocytes on endothelial cells [36]. P2X7 receptor contributes to the regulation of intercellular interactions and to the generation of soluble markers.Elevated levels of CD62L and CD23 in sera have been reported from B-cell chronic lymphocytic leukaemia (B-CLL) patients [37]. ATP-induced L-selectins and CD23 shedding have also been shown to decrease in P2X7 receptor knock-out mice, indicating the pathophysiological role of P2X7 receptor [38]. Involvement of P2X7 receptor in ATP-induced apoptosis is well documented in lymphocytes, monocytes, macrophages, murine thymocytes, and dendritic cells [39,40]. P2X7 receptor activation has been shown to stimulate the activity of intracellular caspases prior to ATP-induced apoptosis. K^+^ efflux through the P2X7 receptor ion channel leads to cell shrinkage and activation of caspase cascades [41].

### P2X7 receptor and regulation of lung surfactant secretion

Lung surfactant is a lipid-enriched substance comprising of 80% glycerophospholipids, 10% cholestrol and about 5-10% proteins. Di-palmitoylphosphatidyl choline (DPPC) is the major glycerophospholipid present in surfactant. The major function of surfactant is to reduce surface tension in the lung. There are 4 major surfactant proteins. SP-B and SP-C are synthesized in endoplasmic reticulumn and further processed by Golgi apparatus. These proteins are stored in lamellar bodies preceeding exocytosis [42]. However, SP-A and SP-D secretes constitutively independent of lamellar bodies [43]. Alveolar epithelial type II cells (AEC II) stores and secrete surfactant. Physiologically, mechanical stretch, labor, and ventilation induce surfactant secretion from AEC II. However, recent experiments suggests that lung distensions rather than systemic changes accompanying hyperventilation (Pco_2_, Po_2_, and pH) increases surfactant secretion [44]. The mechanical stretch of the AEC II during an enhanced inspiration (‘sigh’) is a direct stimulus for surfactant secretion.

The commonly held view of regulated surfactant secretion from AEC II involves cell membrane receptors including β_2_-adrenergic, adenosine A2B, and purinergic P2Y2. ATP, UTP, adenosine, platelet activating factor, LPS, and IL-1β are the known agonists to stimulate surfactant secretion [45]. Stimulation of these receptors ultimately leads to activation of protein kinase A (PKA), protein kinase C (PKC) and calcium and calmodulin kinase (CaMK) and their downstream partners. The purinergic metabotropic receptor, P2Y2, is coupled to the G protein Gq, which stimulates phospholipase C (PLC) and hydrolyzes phosphotidylinositol biphosphate into diacylglycerol (DAG) and inositol triphosphate (IP_3_) [46]. The increase in intracellular Ca^2+^ concentration results in enhanced surfactant secretion [47,48]. Surfactant exocytosis in AEC II is extremely sensitive to perturbations of Ca^2+^. *In vitro,* secretagogues including β_2_-adrenergic agonists (terbutaline), A_2B_ receptor agonists (adenosine), P2Y_2_ receptor agonists (ATP and UTP), PKC activators (phorbol esters) and calcium ionophores (A23187) have been shown to stimulate surfactant exocytosis. Terbutaline and adenosine activate adenyl cyclase which further stimulates PKA-mediated signaling. Moreover, ATP and phorbol esters activate PKC and downstream signaling molecules. The ionophores increase the intracellular calcium concentrations which further activates PKC and CAMK II. Activation of various kinases leads to phosphorylation of various proteins resulting in surfactant exocytosis. However, the mechanism of how the phosphorylation induces secretion is incompletely understood.

### Contribution of AEC I and P2X7 receptor signaling in surfactant secretion

The alveolar epithelium has two specialized epithelial cell types: the terminally differentiated squamous AEC I and the surfactant producing cuboidal AEC II. The AEC I cover 95% of the alveolar surface and form a tight epithelial barrier with the AEC II to facilitate gas and water exchange. Alveolar epithelial cells are closely associated with endothelial cells, stromal fibroblasts, inflammatory cells, and the accompanying extracellular matrix. The function of AEC I has been relatively unexplored because it has been extremely difficult to isolate and culture viable AEC I [49]. Our lab and other labs have developed methods to isolate AEC I [50].

AEC I respond to the forces generated by mechanical ventilation *i.e.* conversion of physical forces on the cell membranes and/or receptors into activation of intracellular signaling pathway leading to Ca^2+^ wave generation. The intracellular Ca^2+^ contributes to integrate signaling in lung epithelium. The mechanisms underlying the coordination of intracellular Ca^2+^ changes in AEC I to neighboring AEC II involve diffusion of ions/second messenger molecules through gap junctions and release of ATP or UTP in the extracellular spaces. This subsequently activates Ca^2+^ signaling pathways in AEC II through purinergic receptors and induces surfactant release. Recent studies using the *in situ* technique confirm that calcium waves passed from AEC I to AEC II result in the release of surfactant from AEC II [51]. Mechanical stimulation of AEC I-like cells in heterocellular culture propagated calcium to neighboring AEC II-like cells mainly via an apyrase-sensitive mechanism, suggesting that ATP is an extracellular mediator of alveolar cell communications [52]. ATP is produced by AEC I in response to mechanical stimulation and in turn triggers surfactant secretion from AEC II [53]. However, the mechanism of ATP release from AEC I has not been established.

P2X7 receptors are specifically expressed in AEC I [29]. The expression of P2X7 receptor couples caveolin-1 as Cav-1 knock-out mice shows reduced P2X7 receptor immunoreactivity in lung [54]. Previously, we have shown that the stimulation of P2X7 receptor in AEC I releases soluble mediator, ATP, which acts in a paracrine fashion on AEC II. Activation of P2Y2 receptors by extracellular ATP increases surfactant secretion from AEC II via a PKC–dependent signaling pathway. Moreover, the paracrine regulation of surfactant exocytosis by P2X7 receptor is a physiologically relevant phenomenon as the P2X7 receptor knock-out mice are less responsive to hyperventilation-induced surfactant release [55]. Therefore, P2X7 receptors in AEC I are an important regulator of surfactant secretion and AEC I and AEC II communications.

### Acute lung injury

Acute Respiratory Disease Syndrome (ARDS) is acute lung injury (ALI) of the alveolar/capillary membrane. ARDS is characterized by permeability pulmonary edema (fluid in the alveolar space) and acute respiratory failure. It is defined as acute respiratory distress with diffuse alveolar infiltrates on chest X-ray, severe hypoxemia (PaO_2_/F_I_O_2_ <200). The etiology of ARDS includes aspiration of gastric contents, pneumonia, smoke inhalation, sepsis, trauma, and drug overdose, multiple transfusions, pancreatitis, and venous air embolism. Sepsis is an important predisposing factor, present in 40% of ARDS patients. Multiple risk factors increase the chance of developing ARDS. ALI is a common complication of mechanical ventilated patients and often synergized the upshots with sepsis background. Mechanical ventilation augments the acute lung injury caused by bacterial products.

Alveolar cells produce a range of proinflammatory cytokines when exposed to bacterial products. Importantly, ATP is a potent candidate to activate the innate immune response. There is a significant degree of purinergic interplay in the prognosis of the ALI pathogenecity. The pathogenesis of the disease can be inferred by concentering on the factors that are responsible for the accumulation of protein-rich and neutrophillic pulmonary edema in the lung and, the mechanisms that impair the removal of pulmonary edema fluid [56]. Inflammatory cells from the lung are the inciting factors in the pathogenesis of ALI. The protein –rich edema fluid in ALI is associated with large numbers of neutrophils, denuded alveolar epithelial cells and proinflammatory markers including cytokines, oxidants procoagulant factors and proteases [57] .

The initial cause of ALI is the lung vascular injury. An increase in lung vascular permeability occurs primarily at the level of lung microcirculation, which in turn results in the accumulation of protein-rich pulmonary edema fluid, even in the presence of normal lung vascular pressur [58]. A sustained loss of normal endothelial barrier function is best described in neutrophil-dependent lung injury [59]. Neutrophil sequestration and activation in the lung microvasculature leads to degranulation and release of several toxic mediators, including ROS, proinflammatory cytokines and procoagulant molecules, resulting in increase of vascular permeability.

Alveolar epithelial injury is another cardinal characteristic of ALI. Although the mechanisms responsible for epithelial injury in ALI are poorly understood, it appears that neutrophil and their products are primarily responsible for the increased paracellular alveolar permeability in ALI. However, neutrophil can cross the alveolar epithelium without affecting the lung epithelial permeability [57,60]. In pathological states the migration of a large number of neutrophils results in increased epithelial injury [56]. Furthermore, the degree of neutrophil activation (priming) by exposure to chemokines and other proinflammatory cytokines seems to play a crucial role in the alveolar epithelial injury as neutrophils crawl into the distal airspaces. Transepithelial migration of neutrophils into the distal airspaces involves three distinct and sequential steps of adhesion, migration and post-migration. Neutrophils adhere to the basolateral epithelial surface by β2-integrins [61,62]. The initial adhesion of neutrophils to the basolateral surface is primarily mediated by CD11b/CD18 molecules [63]. However, CD18-independent transmigration of neutrophils has also been documented. The paracellular route of neutrophil migration to the distal airspaces is also associated with CD47, a cell-surface molecule expressed on epithelial cells and neutrophils [64]. Once they traverse the epithelium and enter the airspaces, neutrophils adhere to the apical surface, where they phagocytize and kill bacteria. The release of toxic intracellular molecules from activated neutrophils induces dissolution of tight junction and necrosis of AEC I. Finally, apoptosis and phagocytosis of inflammatory cells are critical for the resolution of inflammation and mitigation of lung tissue damage.

### P2X7 receptor signaling in acute lung injury

P2X7 receptor knock-out mice are less susceptible to smoke-induced lung inflammation and emphysema. P2X7 receptor expressions increased rapidly in alveolar macrophages, circulating neutrophils and in whole lung tissue following smoke-induced lung inflammation and are blocked by selective intra-pulmonary P2X7 receptor inhibition [65]. Furthermore, P2X7 receptor knock-out mice shows reduced inflammation and lung fibrosis to airway-administered bleomycin. In the presence of ATPγS, a stable P2X7 receptor agonist, lung cell recruitment and matrix remodeling proteins such as matrixmetalloproteinase-9 (MMP-9) and tissue-inhibitor of metalloproteinase (TIMP-1) were increased rapidly. Therefore, P2X7 receptor plays an important pro-inflammatory function in injured alveolus. Delving into the mechanism mediated by P2X7 receptor in injury, we found that P2X7 receptors are involved in soluble Vascular Cell Adhesion Molecule-1 (sVCAM-1) release from AEC I (Unpublished data). sVCAM-1 resembles an important regulatory component of the inflammatory response and is detectable in serum and other body fluids. sVCAM-1 level is elevated in alveolar lining fluid from pneumonia and asthma patients [66,67]. Moreover, our data suggest the metalloprotease, ADAM17 are responsible for VCAM-1 shedding from AEC I surface in a P2X7 receptor-dependent manner. This P2X7 receptor mediated shedding of VCAM-1 from AEC I is crucial for the recruitment of neutrophils in the alveolar space.

## Conclusions

P2X7 receptor plays important roles in immunity, inflammation, bone homeostasis, neurological function and neoplasia. There is increase body of evidence implicating P2X7 receptor in various pathological conditions of pulmonary, cardiac, renal, skeletal muscle and central nervous system (CNS) disorders, where inflammation is the corner stone of these disorders.

The involvement of P2X7 receptor in the pathogenesis of pulmonary emphysema [68], and COPD [69] has been well documented. Pulmonary fibrosis is characterized by inflammation and fibrosis of the interstitium and destruction of alveolar histoarchitecture. Recent animal studies have identified the importance of P2X7 receptor and pannexin-1 complex in IL-1β maturation, inflammation and evolution to pulmonary fibrosis [70].

AEC I play important roles in alveolar barrier protection and fluid homeostasis. Earlier studies indicated that purinergic P2X7 receptors were specifically expressed in AEC I. Although P2X7 receptor-mediated critical signaling pathways in immune cells have been identified, less is known about their functions in alveolar pathophysiology. Several line of evidence suggests that the pro-inflammatory P2X7 receptor signaling are druggable targets in lung inflammatory diseases. However, the mechanism of P2X7 receptor-mediated signaling pathways in alveolus in particular AEC I are not well understood.

**Figure 1:**
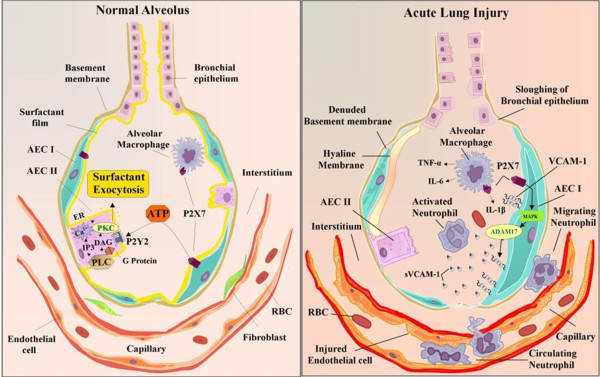
Proposed pathophysiological role of P2X7 receptor signaling in the normal and injured alveolus.

Finally, we propose the following models for P2X7 receptor signaling in alveolus (Figure 1). *(A)* In the normal alveolus, activation of P2X7 receptor in AEC I cell surface leads to ATP release. ATP in the extracellular space activates neighboring AEC II and stimulates surfactant secretion through P2Y2 receptor signaling pathway. *(B)* In the injured lung, there is sloughing of both bronchial and alveolar epithelial cells, denuded alveolar basement membrane. Alveolar macrophages secrete cytokines; IL-1β, IL-6, and TNF-α, that act locally on AEC I. This increases the VCAM-1 expression in AEC I membrane. P2X7 receptor stimulation on AEC I modulate ADAM17 activity through MAPK activation. ADAM17 shed VCAM-1 from the AEC I surface. sVCAM-1 in the alveolar space stimulate neutrophil chemotaxis and sequestrations. Thus, P2X7R resides at the critical nexus of surfactant regulation, cytokine modulation, neutrophil recruitment and inflammation in lung.

## Competing interests

The author has no conflicts of interest to declare.
